# Time-restricted eating on weight loss: implications from the TREATY study

**DOI:** 10.1093/lifemedi/lnac017

**Published:** 2022-06-29

**Authors:** Yan Huang, Deying Liu, Xueyun Wei, Chensihan Huang, Changwei Li, Huijie Zhang

**Affiliations:** Department of Endocrinology and Metabolism, Nanfang Hospital, Southern Medical University, Guangzhou 510515, China; Department of Endocrinology and Metabolism, Nanfang Hospital, Southern Medical University, Guangzhou 510515, China; Department of Endocrinology and Metabolism, Nanfang Hospital, Southern Medical University, Guangzhou 510515, China; Department of Endocrinology and Metabolism, Nanfang Hospital, Southern Medical University, Guangzhou 510515, China; Department of Epidemiology, Tulane University School of Public Health and Tropical Medicine, New Orleans, LA 70112, USA; Department of Endocrinology and Metabolism, Nanfang Hospital, Southern Medical University, Guangzhou 510515, China

Weight loss with calorie restriction is a primary approach for obese patients. However, most trials of dietary approaches to weight loss have reported modest weight loss, and long-term maintenance remains a challenge. Time-restricted eating, an intermittent fasting regimen that involves a shortened eating period within each 24-h period, has gained popularity because it is a simple weight loss strategy to follow, which may sustain circadian rhythms and improve metabolism.

Chronic circadian disruption increases the risks of obesity, metabolic syndrome, hypertension, insulin resistance, inflammation, and dyslipidemia. Recent studies in mice have shown that restricting the time of caloric intake to a window of 8–12 h supports a robust circadian rhythm and reduces caloric intake and imparts pleiotropic physiological benefits, such as reduced adiposity, longer sleep duration, increased endurance, reduced systemic inflammation, improved gut homeostasis, and improvement in relative metabolic biomarkers [1]. Despite these observed benefits in animal model, the applicability of time-restricted eating for human health remains uncertain. Several pilot clinical studies showed that time-restricted eating resulted in reduction over time in the body weight in patients with obesity [2]. In addition, the long-term efficacy and safety of time-restricted eating as a weight-loss strategy are still uncertain. This inspired us to implement a time-restricted eating regimen in obese adults to improve metabolism and reduce weight through sustaining circadian rhythms.

**Figure 1.**
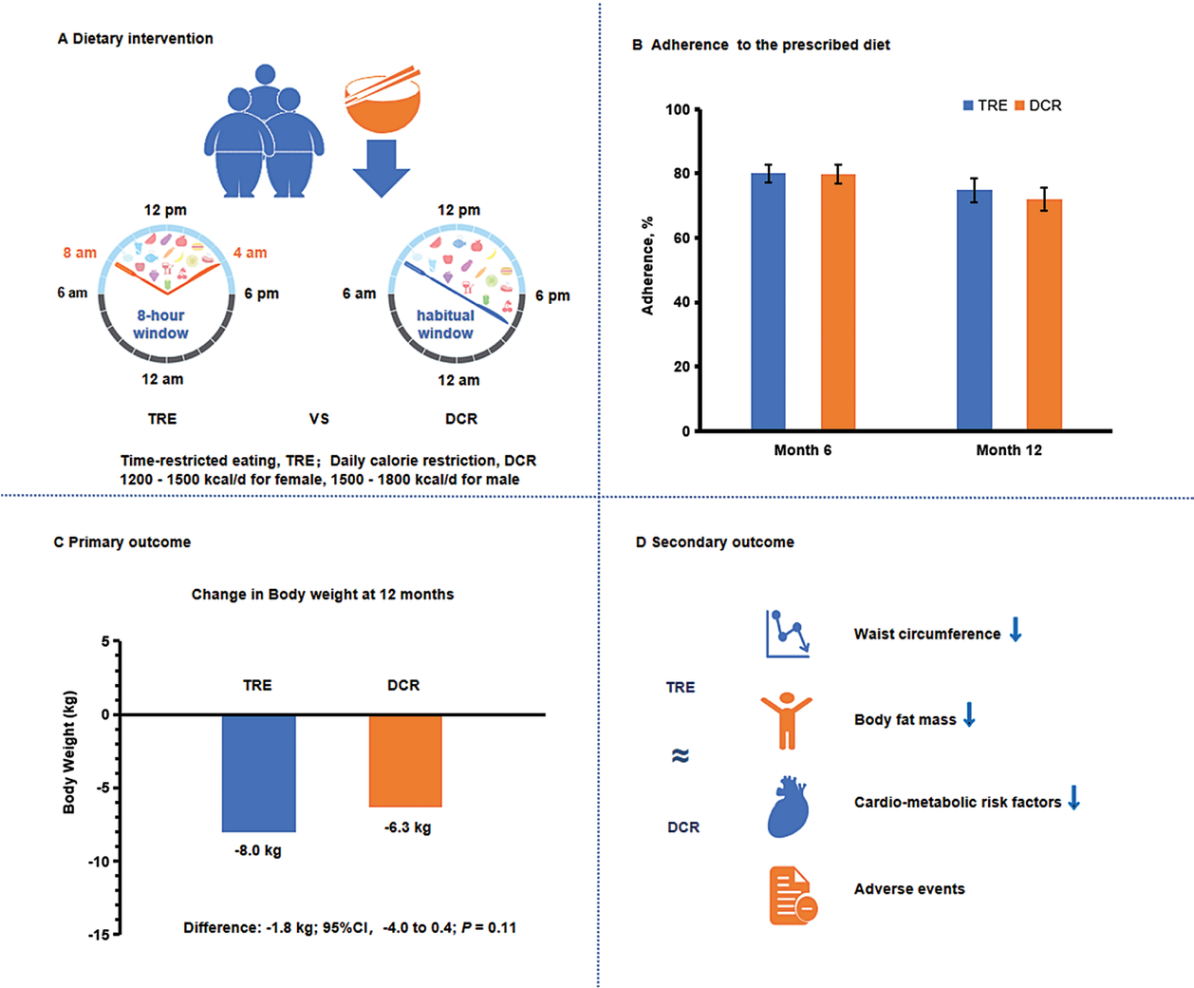
Effects of time restricted rating and daily calorie restriction on weight loss and cardiometabolic risk factors. (**A**) Dietary intervention. (**B**) Adherence to the prescribed diet. (**C**) Primary outcome. (**D**) Secondary outcome. Both 8-h time-restricted eating and daily calorie restriction produced comparable effects on weight loss and metabolic risk factors among obese patients.

TREATY trial was the first randomized clinical trial that assessed the effects of 8-h time-restricted eating with calorie restriction as compared with daily calorie restriction on weight-loss and metabolic risk factors among obese patients [3] (Fig. 1). In this study, 139 adults with a body mass index of 28–45 were assigned to time-restricted-eating group that consumed the prescribed calories within an 8-h period (from 8:00 a.m. to 4:00 p.m.) each day or daily-calorie-restriction group without time restriction. All participants were instructed to follow a diet that represented a 25% calorie reduction from baseline for 12 months (40%–55% of energy from carbohydrate, 15%–20% from protein, 20%–30% from fat). The weight loss from baseline to 12 months was 8.0 kg (95% CI,−9.6 to −6.4 kg; *P* < .001) in the ­time-restriction-eating group and 6.3 kg (95% CI, −7.8 to −4.7 kg; *P* < .001) in the ­daily-calorie-restriction group. The results showed that 8-h time-restricted eating plus calorie restriction was not superior to daily calorie restriction on weight loss (net difference, −1.8 kg; 95% CI, −4.0 to 0.4 kg; *P* = .11). In addition, similar effects with respect to reductions in waist circumference, body fat, visceral fat, blood pressure, glucose levels, and lipid levels were observed in the two groups at the 12-month assessment. No deaths or serious adverse events were reported. In this trial, these results demonstrated that the two weight loss regimens had similar success in obese patients, regardless of whether they reduced their calorie consumption through time-restricted eating or through calorie restriction alone. Furthermore, the results suggest that the time-restricted–eating regimen worked as an alternative option for weight management.

Indeed, time-restricted eating regimen is culturally adopted by Buddhists, a major cultural and religious practice in China. However, the efficacy and safety of this historical practice on obesity management are uncertain. In this 12-month intervention trial, the two dietary regimens had similar success in weight loss and reductions in waist circumference, body fat, visceral fat, blood pressure, and glucose and lipid levels in patients with obesity, regardless of whether they reduced their calorie consumption through time-restricted eating or through calorie restriction alone. Consistently, other results of time-restricted eating also showed that the reduction in insulin resistance, oxidative stress, inflammation, and blood pressure was still observed even when body weight is maintained constant [4]. Therefore, these data support that time-restricted eating is a viable and sustainable approach for the treatment of obesity and metabolic disorder.

It is generally acknowledged that time-restricted eating can improve circadian rhythms and improve metabolic health including glucose and lipid homeostasis, lipolysis and β-oxidation, and inflammation [5]. However, it remained unknown whether these benefits aroused from calorie restriction or time restriction. Experimental studies in mice demonstrated that intermittent fasting can modify circadian rhythms depending on the timing of food availability [6], which highlighted the importance of circadian rhythms on weight loss. Interestingly, some studies suggested that daily fasting length and periodicity have emerged as potential drivers behind beneficial health effects of calorie restriction [7]. Cienfuegos et al. found that time-restricted eating reduced energy intake by 550 kcal per day and produced an ~3% weight loss among 20 obese individuals during 8-week intervention period [2]. In addition, several small clinical studies also showed that time-restricted eating regimen with isoenergetic intake improved metabolic parameters in men with prediabetes [4]. In contrast, Lowe and colleagues documented that time-restricted eating had no favorable benefits with respect to body weight and waist circumference among 116 obese adults over a 12-week period when the strategy of time-restricted eating had no caloric intake deficit [8]. In this clinical trial, these findings highlighted the importance of caloric intake restriction when adhering to a regimen of ­time-restricted eating.

In addition, this trial offered intensive coaching and monitoring to help all participants improve adherence through the use of digital platforms, frequent feedback and attention to dietary quality during the intervention period, which was praised as an important benchmark for a dietary lifestyle intervention [9]. It is well-known that poor adherence to lifestyle intervention is common challenge in the long-term clinical trials. In previous randomized clinical trials of intermittent fasting, a 53.44% dropout rate was observed in the intermittent fasting groups [10], which may decrease the power to detect the hypothesized difference and introduce a possible selection bias between the intervention groups. Based on rigorous design and methods in this trial, nearly 85% of participants completed the trial when delivered with intensive coaching and monitoring, and adherence to the prescribed diets was more than 80% during the 12-month intervention. This trial has several strengths, including a culturally sensitive, ­prescription-based intervention, similar caloric restriction and attention to dietary quality in the two groups, the relatively long duration of the trial, and the high percentage of participants who adhered to the assigned regimen. Of note, it seems difficult to detect a significant difference between the time-restricted eating regimen and daily calorie restriction on weight loss even if the duration of intervention exceeds 12 months, since this trial provided greater than 90% statistical power at significance level of .025.

In conclusion, this trial indicated that the two weight loss regimens that we evaluated had similar success in patients with obesity, regardless of whether they reduced their calorie consumption through time-restricted eating or through calorie restriction alone. A regimen of time-restricted eating was not more beneficial with regard to reduction in body weight, body fat, or metabolic risk factors than daily calorie restriction. These data highlighted the importance of caloric intake restriction when adhering to a regimen of time-restricted eating. Even so, caloric intake restriction through the strategy of time restricted-eating was a viable and sustainable approach for obesity management. Future studies need to elucidate the mechanisms of time-restricted eating on metabolic benefits and assess the effectiveness of time-restricted eating on metabolic diseases in a wide range of populations.
